# Surgical ligation of patent ductus arteriosus in preterm neonates weighing less than 1500g: a 9-year single center experience

**DOI:** 10.1186/s13019-020-01191-2

**Published:** 2020-06-17

**Authors:** Jun Ho Lee, Hyun Ju Lee, Hyun-Kyung Park, Ja-Hye Ahn, Hee Sun Kim, Hyo Jun Jang, Sun Kyun Ro, Hyuck Kim

**Affiliations:** 1 Department of Thoracic and Cardiovascular Surgery, Hanyang University Seoul Hospital, Hanyang University School of Medicine, 222-1, Wangsimni-ro, Seongdong-gu, Seoul, 04763 South Korea; 2 Department of Pediatrics, Hanyang University Seoul Hospital, Hanyang University School of Medicine, Seoul, Republic of Korea; 3 Department of Thoracic and Cardiovascular Surgery, Hanyang University Guri Hospital, Hanyang University School of Medicine, Seoul, Republic of Korea

**Keywords:** Patent ductus arteriosus, Congenital heart disease, Preterm neonates

## Abstract

**Background:**

The aim of this study was to determine the feasibility and outcomes of early surgical ligation in preterm neonates with hemodynamically significant patent ductus arteriosus (HSPDA) and to investigate predictors for surgical treatment after unsuccessful medical management.

**Methods:**

Medical records from the neonatal intensive care unit of Hanyang University Seoul Hospital from January 2010 to December 2018 were retrospectively reviewed. 233 preterm neonates weighing less than 1500g with HSPDA were enrolled in our study. Of these preterm neonates, 134 underwent surgical ligation and were subdivided into the early ligation group (*n* = 49; within 10 days of age) and the late ligation group (*n* = 85; after 10 days of age).

**Results:**

The mean gestational age and birth weight were significantly lower in the patent ductus arteriosus (PDA) ligation group than in the Non-ligation group (*p* <  0.001). PDA ductal diameter > 2.0 mm (*p* <  0.001), low Apgar score at 5 min (*p* = 0.033), and chorioamnionitis (*p* = 0.037) were the predictors for receiving surgical treatment for PDA. Early ligation was significantly associated with a low incidence of culture-proven sepsis (*p* = 0.004), mechanical ventilator time > 4 weeks (*p* = 0.007), necrotizing enterocolitis stage (NEC) ≥ III (*p* = 0.022), and intraventricular hemorrhage (IVH) grade ≥ III (*p* = 0.035).

**Conclusions:**

Early surgical ligation minimizes the adverse effects of HSPDA in predicted preterm neonates who subsequently require surgical treatment for PDA. This result suggests that in preterm neonates weighing less than 1500g with HSPDA that is unresponsive to medical treatment, delayed ductal closure should be avoided to reduce severe NEC, severe IVH, culture-proven sepsis, and facilitate earlier endotracheal extubation.

## Background

Patent ductus arteriosus (PDA) is one of the most common congenital anomalies in premature neonates [1–3] and is associated with increased morbidity and mortality due to acute hemodynamic and respiratory compromise [4, 5]. Although hemodynamically significant patent ductus arteriosus (HSPDA) in preterm neonates has been proven to increase morbidities such as necrotizing enterocolitis (NEC), intraventricular hemorrhage (IVH), bronchopulmonary dysplasia (BPD), heart failure and renal hypoperfusion [5–8], no randomized clinical trial has shown the benefit or efficacy of surgical ligation compared with no PDA ligation in preterm neonates with HSPDA that is refractory to medical treatment [9].

If pharmacologic treatment is contraindicated or fails, surgical ligation can be considered. Surgical ligation can be performed at the bedside in the neonatal intensive care unit (NICU) without a high risk of surgical morbidity or mortality [5]. Although PDA surgical ligation after unsuccessful medical treatment is a definitive treatment, studies on the proper timing of surgery for HSPDA are few [4, 6], and the proper timing of surgical ligation remains controversial.

The goal of this study was to analyze the feasibility of early surgical ligation (within 10 days of age) in preterm neonates with HSPDA and to investigate parameters that would be helpful for deciding on early surgery.

## Methods

### Study populations

Hanyang University Seoul Hospital is a tertiary referral center for patients with a multidisciplinary PDA team in Seoul, South Korea. All medical records of the NICU at our center from January 2010 to December 2018 retrospectively reviewed to identify preterm neonates weighing less than 1500g. Our institutional review board approved this study, and the need for patient consent was waived (IRB number; HYUH 2019–07–004-003).

### Medical treatment

The direction of treatment for PDA was determined by the neonatologist. Most HSPDA patients were initially treated with 2 or 3 cycles of nonsteroidal anti-inflammatory drugs (NSAIDs), such as indomethacin or ibuprofen. The protocol for indomethacin and ibuprofen treatment is described in Table 1. Ibuprofen was initially injected a dose of 10 mg/kg and additionally administered at 5 mg/kg at 24-h intervals up to three cycles, if necessary. Contraindications to NSAID treatment were gastrointestinal bleeding, IVH grade > I, poor urine output (< 0.6 mL/kg/hr), high serum creatinine (> 1.8 mg/dL), high blood urea (> 30 mg/dL), positive disseminated intravascular coagulation (DIC) profiles, or thrombocytopenia (< 60,000 /mm^3^), as in our previous publication [4].

**Table 1:** Protocol for NSAID treatment

Age at 1st dose	1st	2nd	3rd
Indomethacin (mg/kg) ^a)^			
< 48 h	0.2	0.1	0.1
2–7 days	0.2	0.2	0.2
> 7 days	0.2	0.25	0.25
Ibuprofen (mg/kg) ^b)^	10	5	5

*NSAID* nonsteroidal anti-inflammatory drug

^a^At 12- to 24-h intervals. ^b^At 24-h intervals

### Surgical treatment

The decision to refer a preterm neonate with HSPDA to the department of Thoracic and Cardiovascular Surgery for surgical ligation after failure or due to contraindications of medical therapy was performed by a neonatologist and a pediatric cardiologist specialized in echocardiography. All operations were performed at the NICU bedside with the preterm neonate under general anesthesia. A left transaxillary mini-thoracotomy via the 3rd or 4th intercostal space was used as the PDA approach. The ductus arteriosus was ligated using a single medium or medium-large sized titanium Horizon clip (Teleflex Medical, Research Triangle Park, NC, USA). Immediately after the operation, the attending neonatologist checked with echocardiography to determine whether the PDA ligation was successful.

### Endpoints, definitions and follow-up

The PDA ligation group comprised patients who underwent surgical ligation, and the Non-ligation group comprised patients who did not undergo surgical treatment. The PDA ligation group was divided into two subgroups: “early ligation (EL)” and “late ligation (LL)”. As mentioned above, the EL group comprised patients who underwent PDA ligation within 10 days of age, and the LL group comprised patients who underwent surgery after 10 days of age.

The primary outcome of this study was a comparison of mortality between the PDA ligation and Non-ligation groups and the EL and LL groups. Secondary outcomes were factors associated with PDA surgical ligation and postoperative clinical outcomes such as NEC, BPD, IVH (grade ≥ III), sepsis, retinopathy of prematurity (ROP), and periventricular leukomalacia (PVL).

Echocardiography was performed within 3 days of birth, and subsequent echocardiographic parameters were obtained on day 6 (± 1 day), day 9 (± 1 day), and day 14 (± 2 days) to identify symptomatic PDA in preterm neonates. Symptomatic PDA indicated HSPDA, which was defined as a ductal diameter ≥ 1.5 mm, or a ratio of 1.5 or more of the size of the left atrium to the diameter of the aortic root on at least one echocardiography (HD11 Diagnostic Ultrasound Imaging System and Transducers; Philips Ultrasound, Bothell, WA, USA). This definition was based on the description of the previous publication [9]. Associated symptoms with HSPDA classified at our center have been described previously [4].

IVH was classified by Volpe’s grading system [10]. Culture-proven sepsis was defined as the presence of positive findings in one or more blood cultures and clinical symptoms of infection. NEC was defined using Bell’s modified staging criteria [11]. BPD was defined by Jobe and Bancalari’s criteria [12]. The stages of ROP were classified using the international classification of retinopathy of prematurity [13].

### Statistical analyses

Data were expressed as either the mean ± standard deviation or frequency and proportion. Comparisons between groups were performed with chi-square tests and Fisher’s exact tests for categorical variables. Two sample Student’s *t*-tests were used for continuous variables when a normal distribution was found. The Mann-Whitney *U* test was used for variables with skewed distributions. All tests were two-tailed. Logistic regression analysis was used to determine risk factors between the PDA ligation and Non-ligation groups, and clinical morbidities between the EL and LL groups. Receiver operating characteristic (ROC) curves were constructed for the assessment of significant factors associated with PDA surgical ligation. All *p* values of less than 0.05 were considered statistically significant. Statistical analysis was performed using SPSS, version 22.0 (SPSS, Chicago, IL, USA).

## Results

**Fig. 1:**
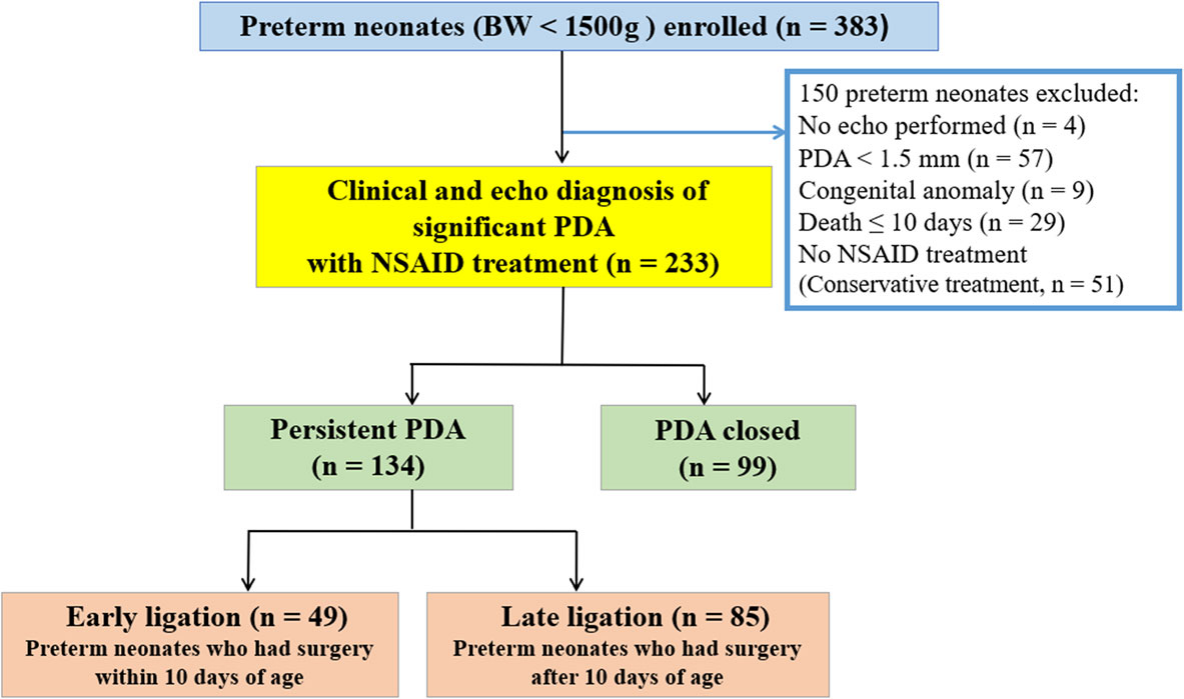
Flow diagram of the study patients. BW, birth weight; PDA, patent ductus arteriosus; NSAID, nonsteroidal anti-inflammatory drug

A total of 233 preterm neonates weighing less than 1500g with HSPDA were ultimately enrolled in our study (Fig. 1). Of these neonates, 134 (57.5%) underwent surgical ligation and were subdivided into the EL group (*n* = 49, 36.6%) and the LL group (*n* = 85, 63.4%). The conditions of 99 neonates improved with medical treatment alone according to the protocol described at Table 1. Nine preterm neonates with congenital anomalies were excluded, including two neonates with congenital tracheoesophageal fistula, one with congenital chylothorax, two with ileal atresia, two with Edwards syndrome, and two with dry lung syndrome.

The baseline characteristics of the patients in the PDA ligation and Non-ligation groups are summarized in Table 2. Regarding maternal characteristics, histologic chorioamnionitis was significantly higher in the PDA ligation group than in the Non-ligation group (*p* = 0.027), while pregnancy-induced hypertension (PIH) was significantly lower in the PDA ligation group than in the Non-ligation group (*p* = 0.031). Regarding neonate characteristics, gestational age (GA), birth weight, length, head circumference, and Apgar scores at 1 min and 5 min were significantly lower in the PDA ligation group than in the Non-ligation group. In contrast, time on a mechanical ventilator or on oxygen therapy, total parenteral nutrition and hospitalization, and age at PDA closure were significantly higher in the PDA ligation group than in the Non-ligation group. Additionally, PDA size (2.13 ± 0.84 mm vs 1.68 ± 0.92 mm, *p* <  0.001) and the proportion of preterm neonates with PDA ductal diameters larger than 2.0 mm (52.2 vs 22.2%, *p* <  0.001) were significantly different between the PDA ligation and Non-ligation groups.

**Table 2:** Characteristics of preterm neonates of the PDA ligation and Non-ligation groups

Variables	PDA ligation (*n* = 134)	Non-ligation (*n* = 99)	*p* value
Maternal characteristics			
Maternal age, years	32.58 ± 4.30	33.29 ± 4.10	0.205
Mother’s education level			0.946
High, n (%)	9 (6.7)	6 (6.1)	
Intermediate, n (%)	88 (65.7)	67 (67.7)	
Low, n (%)	37 (27.6)	26 (26.3)	
GDM	10 (7.5)	10 (10.1)	0.488
PIH	9 (6.7)	16 (16.2)	0.031
PPROM	56 (41.8)	42 (42.4)	> 0.999
Histologic chorioamnionitis	104 (77.6)	63 (63.6)	0.027
Neonate characteristics			
Gestational age, weeks	26.27 ± 2.28	27.37 ± 1.90	< 0.001
Birth weight, grams	882.05 ± 226.61	1053.23 ± 244.76	< 0.001
Length	33.69 ± 4.59	35.96 ± 3.36	< 0.001
Head circumference	24.60 ± 3.10	25.58 ± 2.37	0.010
Cesarean section	98 (73.1)	81 (81.8)	0.157
Male sex	69 (51.5)	41 (41.8)	0.183
Apgar score at 1 min	2.10 ± 1.30	2.73 ± 1.33	< 0.001
pgar score at 5 min	4.58 ± 1.67	5.40 ± 1.55	< 0.001
Antenatal corticosteroids	92 (68.7)	73 (73.7)	0.467
Postnatal corticosteroids	57 (42.5)	31 (31.3)	0.101
Age at PDA closure, days	15.57 ± 8.94	8.10 ± 2.71	< 0.001
Body weight at surgical ligation	911.87 ± 270.16	–	–
IVH	50 (37.3)	43 (43.4)	0.417
IVH grade II	8 (6)	3 (3)	0.241
IVH grade ≥ III	21 (15.7)	17 (17.2)	0.858
PVL	10 (7.5)	9 (9.1)	0.809
Culture-proven sepsis	83 (61.9)	55 (55.6)	0.347
NEC	12 (9.0)	10 (10.1)	0.823
NEC stage ≥ III	41 (30.8)	22 (22.2)	0.180
BPD	74 (55.6)	44 (44.9)	0.112
ROP	80 (60.2)	54 (55.1)	0.501
Days on mechanical ventilator	30.62 ± 18.63	16.20 ± 14.55	< 0.001
Days of oxygen therapy	68.70 ± 52.01	49.41 ± 28.20	0.001
TPN days	44.44 ± 25.64	36.59 ± 21.21	0.014
Hospital days	96.00 ± 40.44	79.89 ± 28.56	0.001
PDA size	2.13 ± 0.84	1.68 ± 0.92	0.001
PDA ductal diameter > 2.0 mm	70 (52.2)	22 (22.2)	< 0.001
Deaths	1 (0.7)	2 (2)	0.576

*PDA* patent ductus arteriosus, *GDM* gestational diabetes mellitus, *PIH* pregnancy-induced hypertension, *PPROM* preterm premature rupture of membranes, *IVH* intraventricular hemorrhage, *PVL* periventricular leukomalacia, *NEC* necrotizing enterocolitis, *BPD* bronchopulmonary dysplasia, *ROP* retinopathy of prematurity, *TPN* total parenteral nutrition

Data are the mean ± SD or number (%)

The baseline characteristics of the patients in the EL and LL groups are summarized in Table 3. Age at PDA closure (8.18 ± 2.14 vs 19.44 ± 8.82 days, *p* <  0.001) and body weight at surgical ligation (847.96 ± 201.73 vs 948.71 ± 297.58 g, *p* = 0.037) were significantly different between the EL and LL groups. The baseline maternal characteristics and preoperative status did not differ significantly between the EL and LL groups. Regarding neonate characteristics, cesarean section was significantly higher in the EL group than in the LL group (*p* = 0.044). In contrast, culture-proven sepsis (*p* = 0.026), NEC stage ≥ III (*p* = 0.030), and time on a mechanical ventilator (*p* = 0.048) were significantly lower in the EL group than in the LL group.

**Table 3:** Characteristics of preterm neonates of the early ligation and late ligation groups

Variables	Early ligation (*n* = 49)	Late ligation (*n* = 85)	*p* value
Maternal characteristics			
Maternal age, years	33.22 ± 5.16	32.21 ± 3.71	0.205
Mother’s education level			0.835
High, n (%)	3 (6.1)	6 (7.1)	
Intermediate, n (%)	31 (63.3)	57 (67.1)	
Low, n (%)	15 (30.6)	22 (25.9)	
GDM	2 (4.1)	8 (9.4)	0.325
PIH	5 (10.2)	4 (4.7)	0.287
PPROM	15 (30.6)	41 (48.2)	0.068
Histologic chorioamnionitis	42 (85.7)	62 (72.9)	0.131
Neonate characteristics			
Gestational age, weeks	26.67 ± 2.52	26.04 ± 2.11	0.123
Birth weight, grams	878.78 ± 216.77	883.94 ± 233.34	0.899
Length	33.10 ± 3.05	34.03 ± 5.27	0.259
Head circumference	24.48 ± 2.30	24.68 ± 3.49	0.719
Cesarean section	41 (83.7)	57 (67.1)	0.044
Male sex	24 (49)	41 (48.2)	> 0.999
Apgar score at 1 min	2.08 ± 1.27	2.11 ± 1.33	0.918
Apgar score at 5 min	4.76 ± 1.58	4.48 ± 1.72	0.367
Antenatal corticosteroids	34 (69.4)	58 (68.2)	> 0.999
Postnatal corticosteroids	17 (34.7)	40 (47.1)	0.205
Age at PDA closure, days	8.18 ± 2.14	19.44 ± 8.82	< 0.001
Body weight at surgical ligation	847.96 ± 201.73	948.71 ± 297.58	0.037
IVH	18 (36.7)	32 (37.6)	> 0.999
IVH grade II	4 (8.2)	4 (4.7)	0.240
IVH grade ≥ III	4 (8.2)	17 (20.2)	0.086
PVL	3 (6.1)	7 (8.2)	0.746
Culture-proven sepsis	24 (49)	59 (69.4)	0.026
NEC	18 (36.7)	24 (28.2)	0.337
NEC stage ≥ III	2 (4.1)	15 (17.6)	0.030
BPD	25 (52.1)	49 (57.6)	0.588
ROP	32 (66.7)	48 (56.5)	0.273
Pneumothorax before ligation	2 (4.1)	2 (2.4)	0.623
Pulmonary hemorrhage before ligation	3 (6.1)	8 (9.4)	0.746
GI hemorrhage before ligation	1 (2.0)	7 (8.2)	0.257
Hypotension before ligation	11 (22.4)	21 (24.7)	0.836
Hypotension after ligation	2 (4.1)	3 (3.5)	> 0.999
Days on mechanical ventilator	26.38 ± 15.31	33.02 ± 19.95	0.048
Days of oxygen therapy	59.88 ± 33.88	74.51 ± 56.89	0.107
TPN days	40.73 ± 21.45	47.41 ± 28.36	0.156
Hospital days	94.53 ± 38.86	95.88 ± 41.52	0.853
PDA size	2.06 ± 0.67	2.16 ± 0.92	0.481
PDA ductal diameter > 2.0 mm	25 (51.0)	45 (52.9)	0.859
Deaths	0 (0)	1 (1.2)	> 0.999

*GDM* gestational diabetes mellitus, *PIH* pregnancy-induced hypertension, *PPROM* preterm premature rupture of membranes, *IVH* intraventricular hemorrhage, *PVL* periventricular leukomalacia, *NEC* necrotizing enterocolitis, *BPD* bronchopulmonary dysplasia, *ROP* retinopathy of prematurity, *GI* gastrointestinal, *TPN* total parenteral nutrition, *PDA* patent ductus arteriosus

Data are the mean ± SD or number (%)

No patients died during their operations. Two neonates required reoperation due to residual flow of PDA, as diagnosed by immediate postoperative transthoracic echocardiography. The residual flow was corrected with an additional metal clip. Three patients died during the postoperative hospital day. In the LL group, one neonate died 100 days after PDA ligation due to NEC. In the Non-ligation group, two neonates died 12 and 17 days after birth, and the cause of death was sepsis. There were no statistically significant differences in mortality rate between the PDA ligation and Non-ligation groups (*p* = 0.576), or between the EL and LL groups (p >  0.999).

**Fig. 2:**
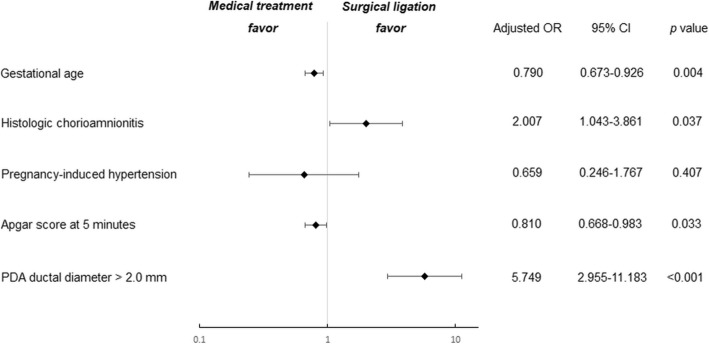
Logistic regression analysis of risk factors associated with PDA surgical ligation. OR, odds ratio; CI, confidence interval; PDA, patent ductus arteriosus. *Odds ratios were adjusted for gestational age, histologic chorioamnionitis, PIH, Apgar score at 5 min, and PDA ductal diameter > 2.0 mm

The logistic regression analysis for factors associated with PDA surgical ligation is presented in Fig. 2. PDA ductal diameter > 2.0 mm (*p* <  0.001), low GA (*p* = 0.004), low Apgar score at 5 min (*p* = 0.033), and histologic chorioamnionitis (*p* = 0.037) remained significant after the analysis was controlled for GA, histologic chorioamnionitis, PIH, Apgar score at 5 min, and PDA ductal diameter > 2.0 mm.

**Fig. 3:**
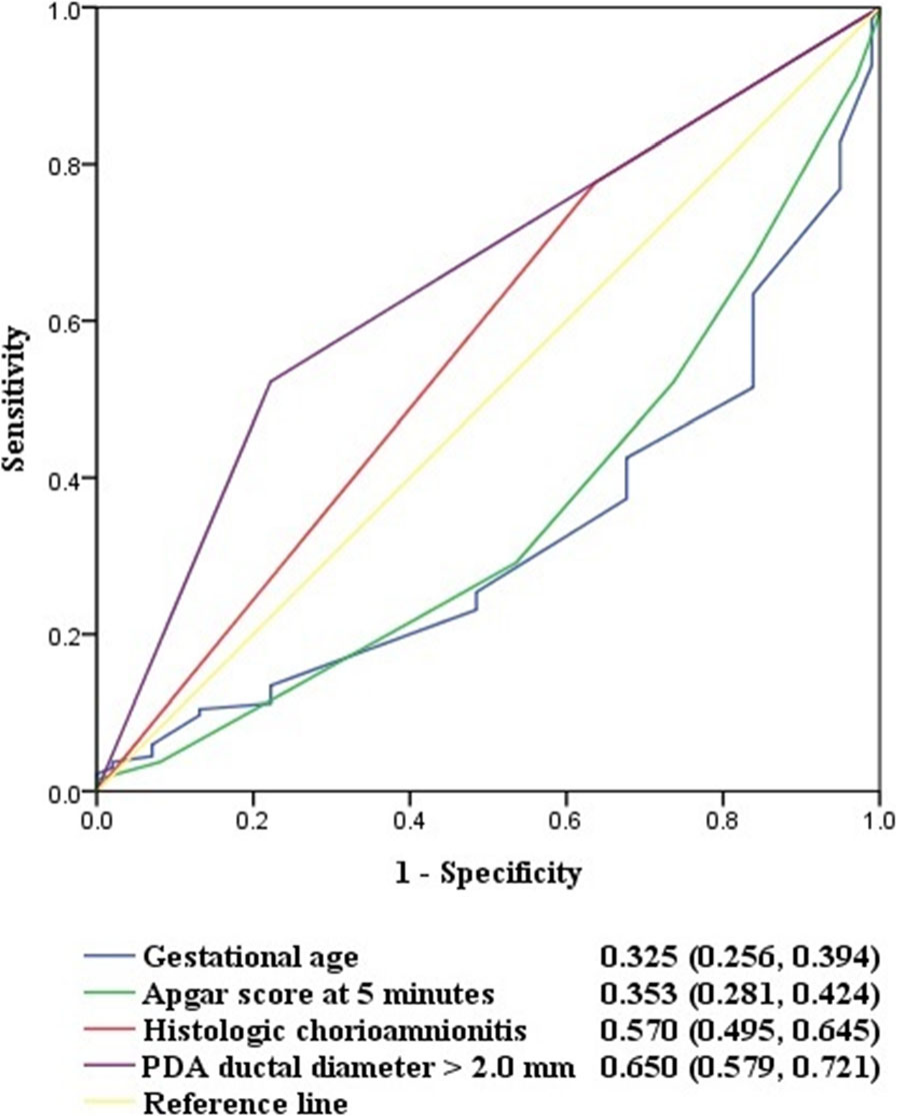
Receiver operating characteristic curve for factors associated with PDA surgical ligation. Figures represent the area under the curve (95% confidence interval). PDA, patent ductus arteriosus

The ROC curve for the ability to predict PDA surgical ligation showed that PDA ductal diameter > 2.0 mm predicted surgical treatment with an area under the curve (95% confidence interval (CI)) of 0.650 (0.579–0.721). Histologic chorioamnionitis showed an area under the curve (95% CI) of 0.570 (0.495–0.645), making it statistically significant in relation to the factors associated with PDA surgical ligation (Fig. 3).

**Fig. 4:**
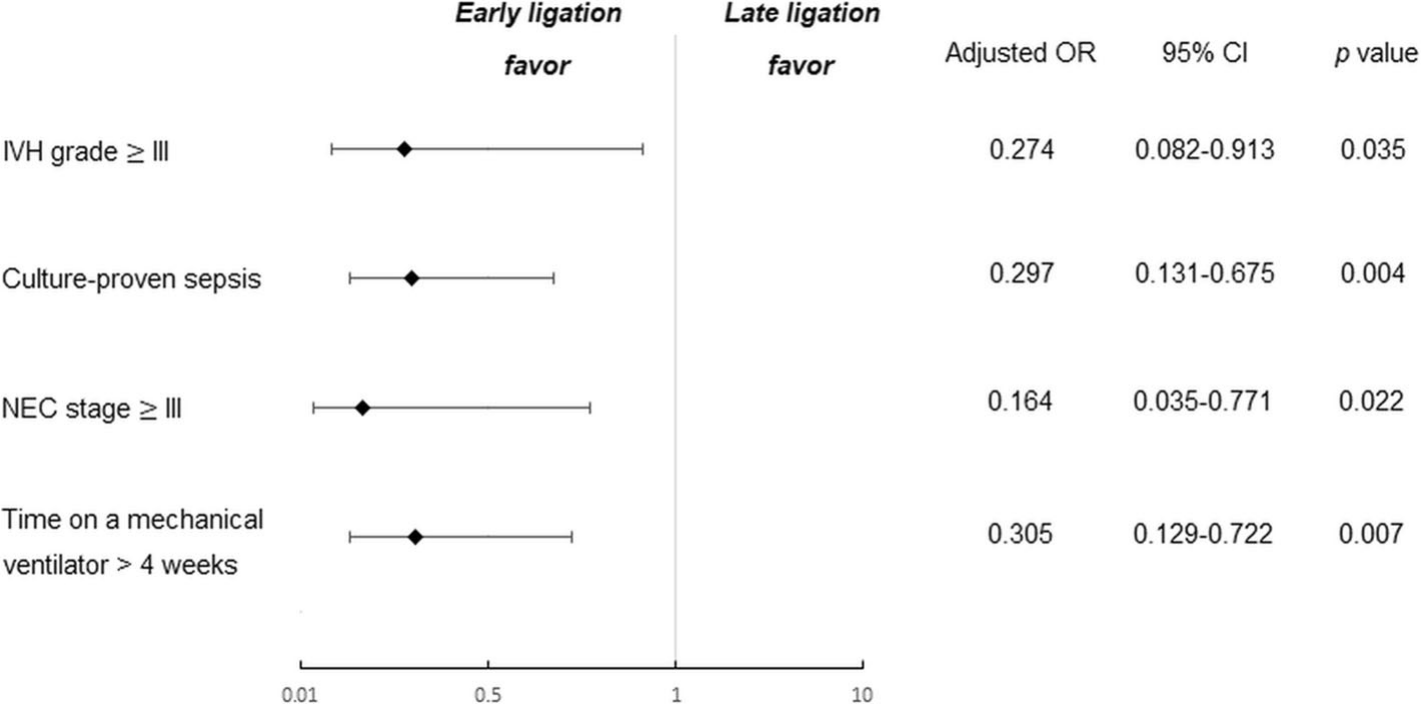
Postoperative clinical outcomes of the patients (early ligation versus late ligation). OR, odds ratio; CI, confidence interval; IVH, intraventricular hemorrhage; NEC, necrotizing enterocolitis. Data are the mean ± SD or number (%).*Odds ratios were adjusted for cesarean delivery, weight on PDA ligation, and PDA ductal diameter > 2.0 mm

The clinical factors that were shown to affect the postoperative clinical outcomes of the patients (EL versus LL) in the multivariate model are shown in Fig. 4. Clinical outcomes with associations with *p* <  0.1 in the univariate analyses were analyzed by multivariate logistic regression with adjustments for cesarean delivery, weight on PDA ligation and PDA diameter > 2.0 mm. After adjustments were made for confounders, EL was found to be significantly associated with a low incidence of IVH grade ≥ III (*p* = 0.035), culture-proven sepsis (*p* = 0.004), NEC stage ≥ III (*p* = 0.022) and time on a mechanical ventilator > 4 weeks (*p* = 0.007).

## Discussion

In this retrospective cohort study of preterm neonates with HSPDA that is unresponsive to pharmacological treatment, there was no statistically significant difference in mortality rate between the PDA ligation and Non-ligation groups. PDA ductal diameter > 2.0 mm, low GA, low Apgar score at 5 min, and histologic chorioamnionitis were related to the need for surgical ligation of HSPDA. Additionally, early surgical ligation was not significantly associated with increased mortality among preterm neonates with HSPDA. The LL group was significantly related to an increased risk of NEC (stage ≥ III), IVH (grade ≥ III), culture-proven sepsis and time on a mechanical ventilator > 4 weeks.

The role of HSPDA surgical ligation in preterm neonates is still controversial. Although surgical treatment can close HSPDA immediately, multiple postoperative comorbidities such as left recurrent laryngeal nerve injury, bleeding, chylothorax, development of coarctation and acute hemodynamic compromise, can be associated with in-hospital mortality [5]. In addition, previous observational studies have demonstrated that surgical treatment is associated with an increased risk of chronic lung disease (CLD), ROP, and neurodevelopmental impairment (NDI) [5, 14–19]. In contrast, a recent observational study demonstrated that there was no significant difference in NDI between the PDA ligation and Non-ligation groups [9]. Furthermore, another previous publication suggested that the preferred option for PDA after unsuccessful medical management should be surgical ligation to avoid prolonged low levels of cerebral saturation [20].

PDA surgical ligation is a viable option that is safe and effective [21], and it can be performed at the bedside in the NICU without transfer to the operating room [5, 22]. In our cohort, no preterm neonates died during their operations, and there was no statistically significant difference in mortality rate between the PDA ligation and Non-ligation groups, and between the EL and LL groups. Although this was a small retrospective study with only three preterm neonates who died and although its statistical power may be limited, this result was shown to be noninferior to those of previous publications [6, 9].

In this study, histologic chorioamnionitis, which is diagnosed by histologic biopsy of the maternal placenta, showed a significant association with factors related to PDA surgical ligation. The role of infection in maintaining the patency of PDA can be considered [23]. Infection may induce the production of cyclooxygenase (COX)-2 and inducible nitric oxide synthetase (iNOS), and together, they increase the production of vasodilatory prostaglandins such as COX-1 and NOS [24]. A preterm neonate born from a mother with chorioamnionitis can have PDA with a persistent opening due to increased levels of vasodilatory prostaglandins and nitric oxide. In addition, clinical factors such as the PDA ductal size and signs reflected in the Apgar score should be emphasized when considering surgical intervention to improve clinical outcomes.

In preterm neonates with HSPDA, it is important to determine the optimal timing of surgical ligation [21, 25]. Our results showed that the EL group was associated with lower odds of severe NEC and IVH than the LL group. This may be explained by the diastolic steal of systematic circulation through HSPDA, which can induce intestinal ischemia resulting in NEC, renal hypoperfusion, and a reduction in the blood flow rate in the middle cerebral artery [26] and increase the risk of IVH [27]. In our previous publication, early surgical ligation had the benefit of reducing the incidence of NEC and improving feeding intolerance [4]. The difference between this previous study and the current observational study is embodied, and there is a significant difference in severe NEC (stage ≥ III) between the EL and LL groups.

Additionally, sepsis with increased serum levels of inflammatory mediators or prostaglandins can be associated with smooth muscle relaxation of the ductus arteriosus [28]. Thus, as previously mentioned, the role of infection in maintaining the patency of PDA can be considered [23]. EL may reduce the duration of infection exposure and can be expected to minimize the risk of sepsis.

Prolonged patency of PDA increases pulmonary circulation that can be injurious to the capillary endothelium and stimulate an inflammatory cascade that results in pulmonary edema, CLD development, and increased ventilator support [29]. EL may diminish the period of exposure to HSPDA [30] and can decrease pulmonary edema [29] and facilitate earlier endotracheal extubation [9].

This study has several limitations. First, this was a small retrospective study with only 233 patients, and a randomized and prospective trial could not be performed. Thus, the statistical power of the population may be limited. Second, all operations were performed by one cardiac surgeon with a high level of experience in pediatric heart surgery. One goal of future research is to perform a large prospective multicenter study with long-term and close follow-up of preterm neonates with HSPDA that is refractory to pharmacological treatment.

## Conclusions

Early surgical ligation minimizes adverse effects of HSPDA in predicted preterm neonates who subsequently require surgical treatment for PDA. This result suggests that in preterm neonates weighing less than 1500g with HSPDA that is unresponsive to medical treatment, delayed ductal closure should be avoided to reduce severe NEC, severe IVH, culture-proven sepsis, and facilitate earlier endotracheal extubation. Avoidance of delayed surgical ligation after failure of pharmacologic closure may have beneficial outcomes in terms of postnatal morbidity among preterm neonates with risk factors in relation to HSPDA surgical ligation.

## Data Availability

Please contact the corresponding author for data requests.

## Abbreviations

HSPDA: Hemodynamically significant patent ductus arteriosus
NEC: Necrotizing enterocolitis
IVH: Intraventricular hemorrhage
PDA: Patent ductus arteriosus
BPD: Bronchopulmonary dysplasia
NICU: Neonatal intensive care unit
NSAID: Nonsteroidal anti-inflammatory drug
DIC: Disseminated intravascular coagulation
EL: Early ligation
LL: Late ligation
ROP: Retinopathy of prematurity
PVL: Periventricular leukomalacia
ROC: Receiver operating characteristic
PIH: Pregnancy-induced hypertension
GA: Gestational age
CI: Confidence interval
CLD: Chronic lung disease
NDI: Neurodevelopmental impairment
COX: Cyclooxygenase
iNOS: Inducible nitric oxide synthetase
